# Structural Basis for Modulation of Quality Control Fate in a Marginally Stable Protein

**DOI:** 10.1016/j.str.2015.04.015

**Published:** 2015-07-07

**Authors:** Kelly P. Brock, Ayelet-chen Abraham, Triana Amen, Daniel Kaganovich, Jeremy L. England

**Affiliations:** 1 Computational and Systems Biology, Massachusetts Institute of Technology, 77 Massachusetts Avenue, Cambridge, MA 02139, USA; 2 Department of Cell and Developmental Biology, Hebrew University of Jerusalem, Edmond J. Safra Campus, Givat Ram, Jerusalem 91904, Israel; 3 Alexander Grass Center for Bioengineering, Hebrew University of Jerusalem, Edmond J. Safra Campus, Givat Ram, Jerusalem 91904, Israel; 4 Physics of Living Systems Group, Department of Physics, Massachusetts Institute of Technology, 400 Tech Square, Cambridge, MA 02139, USA

## Abstract

The human von Hippel-Lindau (VHL) tumor suppressor is a marginally stable protein previously used as a model substrate of eukaryotic refolding and degradation pathways. When expressed in the absence of its cofactors, VHL cannot fold and is quickly degraded by the quality control machinery of the cell. We combined computational methods with in vivo experiments to examine the basis of the misfolding propensity of VHL. By expressing a set of randomly mutated VHL sequences in yeast, we discovered a more stable mutant form. Subsequent modeling suggested the mutation had caused a conformational change affecting cofactor and chaperone interaction, and this hypothesis was then confirmed by additional knockout and overexpression experiments targeting a yeast cofactor homolog. These findings offer a detailed structural basis for the modulation of quality control fate in a model misfolded protein and highlight burial mode modeling as a rapid means to detect functionally important conformational changes in marginally stable globular domains.

## Highlights

• A novel mutation of VHL displays significantly enhanced stability in yeast
• The yeast homolog of elongin C helps stabilize this mutated protein in vivo
• Both modeling and experiment can help characterize protein marginal stability

## Graphical Abstract

**Figure:**
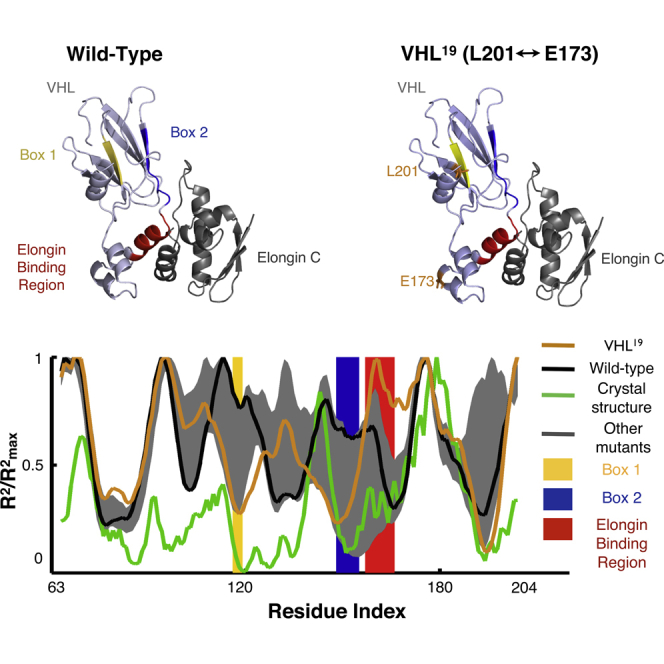

## Teaser

Brock et al. present a novel mutation of VHL, a marginally stable protein that displays significantly enhanced stability in yeast. The structural basis for this effect, which was determined computationally and experimentally, is an increased ability to interact with a homologous cofactor present in yeast.

## Introduction

To function properly in the cell, a globular protein chain typically must remain folded into a specific conformation or set of conformations known as its native state. A primary determinant of how a globular protein folds is its amino acid sequence, which fixes the pattern of internal and external forces that act on the polypeptide chain in the aqueous environment. When a protein cannot reach or cannot maintain its native conformation in the cell, it is considered to be in a misfolded state, which is often accompanied by a loss of cellular function. Misfolded proteins also exhibit a marked tendency to associate non-specifically and sometimes form potentially cytotoxic aggregates if left to accumulate in the crowded intracellular environment (Amen and Kaganovich, 2015; Chiti and Dobson, 2006, 2009; Luby-Phelps, 2000). A large range of globular proteins have been shown to form amyloid fibrils under partially denaturing conditions (Chiti and Dobson, 2009). Mutations or stochastic processes that lead to protein misfolding and/or aggregation have been implicated in proteinopathies such as Huntington's, Alzheimer's, Parkinson's, and the prion-based Creutzfeldt-Jakob disease, emphasizing a need to better understand the cellular response to protein misfolding in the context of the physical driving forces that govern how an amino acid sequence can reach its native structure (Mulligan and Chakrabartty, 2013).

The protein quality control (PQC) machinery consists of the cellular pathways linked to protein folding and misfolding. Chaperone proteins, a key component of PQC systems, can either assist a misfolded protein in refolding or target incorrect conformations for destruction through the ubiquitin proteasome pathway or autophagy (Kim et al., 2013). Despite the prevalence of PQC machinery across organisms, many aspects of the system are not well understood. Misfolded proteins are able to reach a wide variety of different non-native states, and the PQC must be able to recognize this diverse group of conformations either to assist refolding or to target them for destruction before the misfolded polypeptide can detrimentally affect the cell. Hsp70, a pleiotropic heat-shock-induced chaperone conserved from bacteria to eukaryotes, has been shown to recognize exposed hydrophobic sites, particularly short (∼5–7 residues) hydrophobic sequences flanked by positively charged amino acids (Marcinowski et al., 2013; Rüdiger et al., 1997). The eukaryotic chaperonin TRiC, on the other hand, has eight distinct subunits, which are each capable of recognizing distinct motifs in a variety of substrates, with mutations in different subunits leading to different cellular phenotypes (Amit et al., 2010; Spiess et al., 2006). Because the outcome of the PQC triage decision must ultimately depend on the structure of a protein, investigating the role of small conformational perturbations in the sensitivity of a misfolded protein to the QC machinery can indicate how the fate of a PQC substrate is modulated for typical substrates.

Marginally stable proteins, which can misfold easily and exist at a tipping point between stable conformations and PQC-targeted misfolded variants, have been used as an experimental mechanism to explore PQC substrate recognition and subsequent refolding and degradation pathways. The human von Hippel-Lindau (VHL) protein is one such example that is particularly susceptible to incorrect folding. This model misfolding protein forms part of an E3 ubiquitin ligase complex that targets molecules like HIF-1^α^ for degradation, and has been cataloged in depth because hundreds of mutant forms have been linked to cancer pathways in humans (Nordstrom-O’Brien et al., 2010). The first ∼60 residues of VHL remain disordered in the native state; however, the 213-residue protein as a whole must traverse a distinct folding pathway in vivo, including interacting with chaperones such as Hsp70 and TRiC and binding with its cofactors elongin B and elongin C, to achieve a state resistant to cellular degradation (McClellan et al., 2005; Schoenfeld et al., 2000). For TRiC, two short motifs in the VHL sequence (Box 1 and Box 2) have been shown to be necessary and sufficient for TRiC binding to VHL in yeast (Feldman et al., 2003). When folded correctly in complex with its cofactors, the non-disordered region adopts a well-defined tertiary structure; however, the protein adopts a molten globule state without its binding partners in vitro that consists of a partially collapsed state with some secondary structure but no tertiary structure (Sutovsky, 2004). This molten globule state indicates that VHL has difficulty achieving its native state without interactions with other proteins. Perturbations to the system, including mutations to VHL, often lead to a misfolded or otherwise non-functional version of the protein in vivo (Hansen et al., 2002; Knauth et al., 2006). When VHL is introduced into non-native systems like *Saccharomyces cerevisiae* or *Escherichia coli*, where it does not exist naturally, the protein cannot achieve a biologically stable state and in yeast is quickly degraded by the cell (Kaganovich et al., 2008; Melville et al., 2003; Sutovsky, 2004; Weisberg et al., 2012; Ogrodnik et al., 2014). Since VHL is a protein with a typical state that is poised between adequate folding and being targeted for destruction in yeast, it is ideal for use as a probe of how different folds (or misfolded variants) can lead to diverse outcomes through PQC pathways.

One of the persistent difficulties in understanding the physical mechanisms of protein misfolding and subsequent PQC interactions is that almost by definition, misfolded proteins are not amenable to conventional methods of structural characterization. Protein chains that adopt many different conformations cannot be crystallized easily, and aggregation-prone proteins are difficult to solubilize for in vitro characterization. Thus, in examining the effects of different mutations on a marginally stable protein like VHL, a computational model that could give insight into the resulting structural changes could offer a new and much needed perspective on the connection between sequence, structure, and recognition by PQC machinery for a large number of sequences. Recently, we developed a phenomenological model to predict tertiary structural information from sequence alone in globular proteins, which has shown promise as a method of computationally exploring the allowed conformational space of fluctuating protein folds (England, 2011). The burial trace is computed by minimizing an energy function consisting of the hydropathies of each residue and the stretching between neighbor amino acids, subject to steric constraints. The calculation generally takes less than a second to run for short sequences, and adding noise to the parameters of the system can generate an ensemble of amino acid burial patterns for a given protein sequence, which can be used to investigate the variability in structures that a protein can adopt. The rapidity of this model in determining structural information makes it an excellent candidate for probing large numbers of potential mutations of marginally stable proteins to understand PQC response to different conformations in silico and to guide in vivo experiments. This analysis could also shed light on a possible functional role for marginal stability, which may enable sensitive modulation of expression through qualitative transitions in conformational state.

To investigate the link between the underlying biophysics of protein folding and the PQC fate of a model misfolded protein, the burial mode model was used to investigate the folding characteristics of the human VHL tumor suppressor protein. Burial traces were calculated to predict exposed residues for the lowest energy conformations of different mutations of VHL, 20 of which were generated experimentally and tested for their degradation properties in vivo. One of these mutations had markedly and consistently higher levels of VHL present at steady state. Through the use of burial mode analysis, the structural basis of its enhanced ability to persist in the cell was characterized. Our findings confirm that VHL sits on a structural tipping point in sequence space, where a single mutation can lead to a qualitative shift in folding stability, which leads to an altered quality control outcome. Not only do these results highlight the power of a new computational model in gaining elusive information about the structure of intrinsically disordered proteins, they also raise the possibility that such proteins may generally be poised to exhibit strong sensitivity to mutation in vivo, where small perturbations can lead to large differences in the amount of folded protein that survives PQC supervision.

## Results

Our initial goal was to see if we could use computational modeling of the VHL protein to design mutations that would alter its misfolding and degradation. Since VHL is a human protein, it lacks its elongin binding partners when expressed in yeast and thus is quickly degraded. We began by using burial mode analysis to design mutations of VHL that would be expected to reduce the misfolding propensity of the protein and thus slow the degradation of the protein in the yeast cytosol.

### Description of Biophysical Model

**Figure 1:**
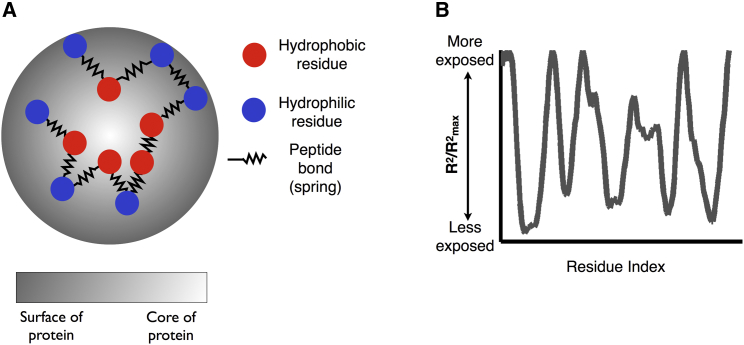
Overview of Burial Mode Modeling (A) An amino acid chain can be modeled as points connected by springs representing peptide bonds. An energy function including costs for stretching neighbor residues apart and putting hydrophobic residues on the outside of the protein can be minimized under our desired steric constraints to predict low energy burial patterns. (B) An example of a burial trace, plotting a measure of the distance of an amino acid from the center of mass of the protein in its lowest energy conformation versus the index of that amino acid in the protein sequence.

Our previous work presented a phenomenological framework to estimate the burial trace or the distance of each amino acid from the center of mass in the lowest energy fold of a globular polypeptide chain. This burial trace predictor assumes that each amino acid behaves like it is connected by a spring to its neighbor residues, simulating peptide bonds (Figure 1). These springs contribute to the overall energy for the system, along with the energetically favorable terms of putting hydrophilic residues far from the center of the protein and burying hydrophobic ones. This energy function can then be minimized through linear optimization under geometric constraints, specifically to ensure that the individual peptides are not clustered into a small volume that would lead to prohibitive steric clashing in a physical scenario. The calculation generally takes less than a second to run for short sequences and results in a prediction of the distance of each residue from the center of the protein in its lowest energy state, called the burial trace.

The relatively small size (213 residues) of VHL and the predominance of α-helical structure in its non-disordered region make it a good candidate for use of the burial mode model. As a first attempt at modeling VHL stability, all $\left(\begin{array}{c} 213\\ 2 \end{array}\right)$ possible VHL pair-swap mutations (where amino acids at two different locations in the sequence are switched, which preserves overall residue frequencies for the sequence) were ranked according to a parameter called structural variability. The hypothesis behind our procedure was that VHL mutants that were less capable of fluctuating between structurally different conformations at low energy (i.e. at energies comparable with the thermal energy scale kT above the energy of the optimal [native] configuration) were less likely to be recognized as misfolded by the quality control system. Accordingly, for each pair-swap mutation, we first calculated the burial trace that was maximally different from the optimal lowest energy trace at fixed, low energy. Then, we ranked the mutations, and selected those ten for which this maximized difference was smallest. This procedure yielded ten least structurally variable mutants to test experimentally. We also selected a control group of ten mutations that were randomly chosen with uniform probability over all possible pair-swap mutations. The complete set of mutations is listed in Table 1.

**Figure 2:**
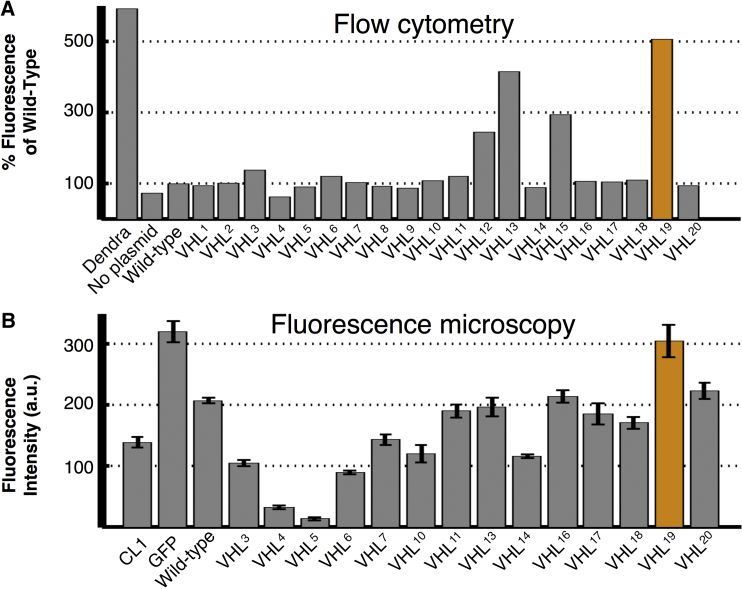
Analysis of VHL Mutant Stability by Flow Cytometry and Fluorescence Microscopy (A) Fluorescence measurements by quantitative flow cytometry for all experimentally created VHL mutations and normalized to fluorescence of the wild-type sequence. The orange entry indicates the most stable mutant, VHL^19^ (L201-E173), in both panels. (B) Selected mutants were also introduced to *S. cerevisiae* cells and expressed endogenously as a GFP fusion construct, and observed directly by fluorescence microscopy with results normalized to GFP levels without VHL. Error bars for all sections represent SE. See also degradation curves in Figure S1. a.u., arbitrary units.

All 20 mutations were then created in an *S. cerevisiae* constitutive plasmid with an attached fluorescent Dendra2 tag and transformed into budding yeast cells, which contain neither native VHL protein nor the human elongin cofactors. If the VHL protein were able to adopt a fold that is resistant to PQC degradation, then the attached fluorescent tag would also be preserved and observable. Fluorescence was detected by using quantitative flow cytometry (Figure 2A). A subset of these mutants were then tagged with GFP and integrated into the genome under the control of a galactose-operated promoter, and their expression level was quantified by fluorescence microscopy at steady-state levels (Figure 2B).

Despite how they were chosen, the first ten non-randomly chosen mutations exhibited lower overall fluorescence than the ten randomly chosen control mutations. This result could be interpreted to mean that either the predictions of the burial mode model were wrong or else that our chosen criterion for enhanced VHL folding (low predicted structural variability) was the wrong one. Had the random control set behaved no differently from the designed mutants, the trail would have gone cold at this point. Surprisingly, however, two mutations in the random control set achieved greater than a 3-fold increase over the wild-type sequence in the flow cytometry data. The mutant V155-G19 (henceforth referred to as VHL^13^), which demonstrated 70% of the fluorescence of the Dendra2 tag alone, changed a hydrophobic residue in the Box 2 region (spanning residues 148–155), which mediates the interaction of chaperonin TRiC with VHL and replaced it with the smaller and more flexible glycine, which is normally located in the disordered N-terminal region. This mutation was distinct in our set because it was the only one that directly affected a known chaperonin binding site, which provided a natural rationale for its altered degradation.

The mutant VHL^19^, L201-E173, however, exhibited the highest level of fluorescence, with a 5-fold increase in steady-state levels compared with the wild-type sequence and ∼85% of the baseline Dendra2 level. The high steady-state levels of this mutation were further confirmed in the fluorescence microscopy. The biophysical characteristics of the residues swapped were dissimilar, with the hydrophobic leucine at residue 201 changing into a negatively charged glutamic acid (normally at residue 173) and vice versa. Although both mutations occurred in the region that adopts a well-defined conformation during correct folding, neither was in a known binding site for cofactors or chaperones, raising the possibility that an allosteric response could explain the structural change necessary to evade PQC degradation systems.

Past work has established several interaction sites in the well-folded region of VHL, including two short motifs called Box 1 (residues 114–118) and Box 2 (residues 148–155), which are known to bind to chaperonins (Feldman et al., 2003), and a short binding motif for cofactors of VHL elongin B and elongin C. A BLAST search for VHL cofactors elongin B and elongin C also revealed that although elongin B does not have a counterpart in yeast, a homolog of elongin C is present. Furthermore, this homolog has been shown to bind to a VHL fragment containing residues from 157 to 171, which contains the known elongin B/C binding site, in vitro (Botuyan et al., 2001). Since elongin B and C stabilize VHL in human cells, one testable hypothesis is that burying the chaperonin interaction sites (and therefore possibly protecting VHL from chaperone-assisted degradation) and exposing the elongin interaction site could help explain why certain mutations caused the protein to resist degradation in the cell.

**Figure 3:**
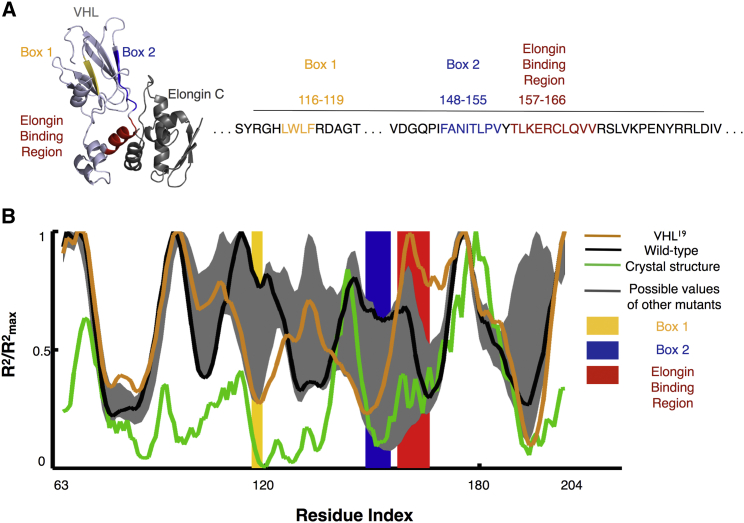
Burial Mode Analysis of VHL Mutants (A) The crystal structure of parts of VHL can be obtained when the protein is bound in complex to cofactors like elongin C, which binds to VHL between residues 157 and 166. VHL also is known to interact with chaperones in regions called Box 1 and Box 2. (B) The VHL^19^ mutation (orange line) is predicted to bury its Box 1 and Box 2 regions, suggesting a conformation that is closer to the form the protein takes when stably bound as part of the VBC complex (green line). The elongin interaction site of the mutation is also predicted to be highly exposed compared with both the wild-type (black line) and the other experimental mutations (gray envelope). Locations of specific mutations are shown in Figure S2.

To explore how mutations could affect conformations, particularly in understanding exposure patterns of different interaction sites, the burial trace of each experimentally created mutant was computed using the burial model and compared with the actual burial trace calculated from the crystal structure of the well-folded region. Since the basic burial trace model works best on α-helical structures, each input sequence was truncated to residues 63–204, corresponding to the region that can be crystallized (PDB: 1VCB) in an attempt to improve accuracy within the region that can adopt a well-defined folding state (Stebbins et al., 1999). Figure 3 shows the predicted burial traces for the wild-type and VHL^19^ sequences, along with maximal and minimal predicted burial values at each residue for all 20 experimentally created mutations. The experimental burial trace obtained directly from the crystal structure of wild-type VHL (PDB: 1VCB chain C) is also shown. The crystal structure burial trace shows a distinctive burial of the Box 1 and Box 2 regions, suggesting that TRiC uses these regions to recognize the failure of the protein to fold natively. The elongin binding site, meanwhile, is moderately exposed compared with Box 1 and Box 2 in the native conformation.

**Figure 4:**
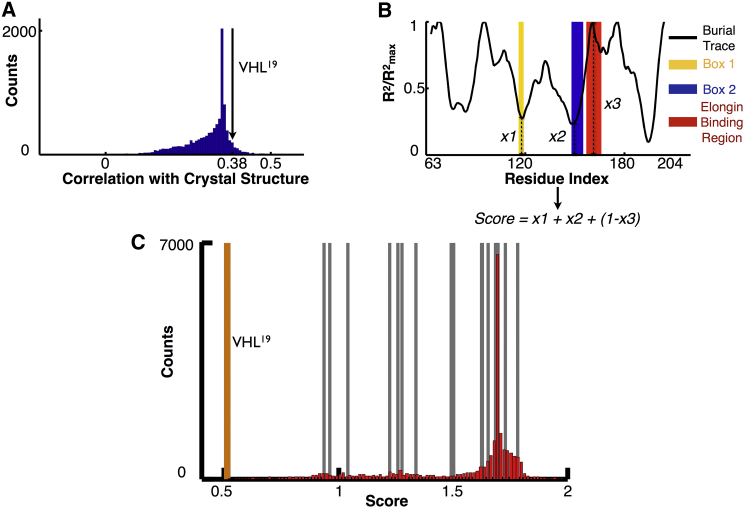
Burial Pattern of VHL^19^ Is Distinct from the Other Mutated Sequences (A) Compared with all possible pair-swap mutations in the region of VHL that can be crystallized (residues 63–204), VHL^19^ (black arrow) is predicted to have a higher burial pattern correlation with the crystal structure than 93.82% of all other possible mutations. (B) An illustration of how scores were generated for each possible mutant, where the smallest score would correspond to maximal burial of the Box 1 and 2 regions and maximal exposure of the elongin interaction site. (C) Each possible pair-swap mutation in the region (63,204) was scored according to Figure 4B. VHL^19^ has the lowest (best) score out of ∼10,000 mutations. The locations of the other experimental mutants in the histogram are indicated by the gray lines, with VHL^19^ marked by the orange line.

Of all the experimentally tested mutants, the well-stabilized VHL^19^ mutation (Figure 3, orange line) is predicted to have the most buried Box 1 and Box 2 regions, similar to how TRiC is able to bury these motifs in achieving its native conformation in human cells. Furthermore, the elongin interaction region of the VHL^19^ mutation was distinctive in that the burial mode model predicted that it was one of the most exposed regions in the entire sequence. This property of burying known chaperone interaction sites and exposing the cofactor binding site is unique among the other mutant and wild-type sequences (Figure 3, gray envelope and black line, respectively), and is more similar to the known native conformation of the protein derived from its crystal structure. To test the significance of this finding, we also calculated each possible pair-swap mutation in the region of VHL that can be crystallized (residues 63–204) and compared their burial traces by Pearson correlation with the burial trace of the crystal structure. Only 6.18% of possible mutations in this region had a higher correlation with the crystal structure than the VHL^19^ mutant (Figure 4A).

The two stabilizing mutants were unique in that they affected chaperone binding sites, either through direct mutation, as in the case of VHL^13^, or through causing the burial of these sites to be more energetically favorable, as in VHL^19^. The other distinctive component of the VHL^19^ burial trace, however, was its extreme exposure of its elongin cofactor binding site. We hypothesized that VHL^19^ evades degradation by combining reduced chaperonin interaction with increased stabilization through interaction with the elongin cofactor. Thus, we scored all possible pair-swap mutations by ranking directly how well the Box 1 and Box 2 chaperonin interaction regions were buried and the elongin interaction site was exposed based on the predictions of the burial model. This score can be obtained from a mutated sequence by calculating the sum of the predicted distances of the most buried residues in the Box 1 and 2 regions from the center of mass of the protein and adding this subscore to the predicted distance of the maximally exposed residue in the elongin interaction region from the most exposed residue of the entire protein (Figure 4B). The smallest score would correspond to Box 1 and Box 2 being located at the center of the protein and the elongin interaction site being on the most external part of the surface of the protein, which would in turn correspond to the hypothetically best way of evading degradation by achieving greater folding stability in the cell.

Amazingly, according to this scheme, VHL^19^ turned out to have the lowest (most optimal) score of ∼10,000 possible mutations (Figure 4C). The location of the other experimentally determined mutants is also shown, indicating a large gap in scores between mutant VHL^19^ and the rest of the experimental sequences. The scores of the rest of the sequences, however, did not correlate with their fluorescence levels, indicating that this may be a binary effect observed only at the extreme achieved by VHL^19^.

**Figure 5:**
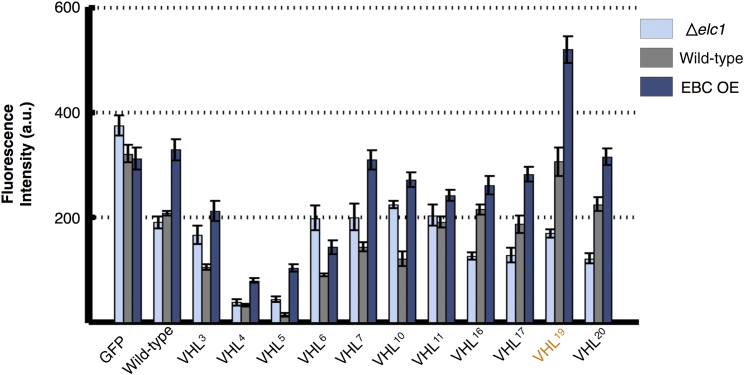
Analysis of Mutant VHL Interaction with Elc1 through Deletion and Overexpression GFP constructs with VHL were expressed in wild-type *S. cerevisiae* (gray, center in grouping), a knockout strain for the yeast homolog of elongin C Δelc1 (light blue, leftmost in grouping), and in the EBC OE strain, which introduced and overexpressed human versions of elongin B and C (dark blue, rightmost in grouping). Although expressing VHL^19^ in the Elc1 knockout strain decreased the levels of the mutant VHL to that of wild-type VHL, the presence of human elongin B and C markedly increased VHL^19^ levels. Error bars represent SE in all sections. Degradation curves for the wild-type sequence under both EBC OE and knockout conditions are also shown Figure S1. a.u., arbitrary units.

The homolog of elongin C, Elc1, is not an essential protein in *S. cerevisiae*, so to test our hypothesis that the VHL19 mutation helped stabilize the protein by decreasing chaperone interactions and/or interacting with Elc1, the mutated VHL sequences were transformed into an Elc1 knockout of yeast. Fluorescence microscopy was again used to measure persistence of the VHL mutant protein in the cell (Figure 5). The stable expression of VHL^19^ in the knockout strain Δ*elc1* was decreased to that of the wild-type, indicating that Elc1 could be helping to stabilize this mutation and lending credence to the findings of the burial model. A plasmid containing human elongin B and C, known to help stabilize VHL in humans, was then transformed to cells expressing the integrated VHL mutants and constitutively expressed (EBC OE strain). The presence of the natural cofactors of VHL further stabilized the VHL^19^ mutant by ∼70%, providing more evidence that the cofactor elongin homolog directly contributes to the exceptional extra stability of the VHL^19^ mutant.

**Figure 6:**
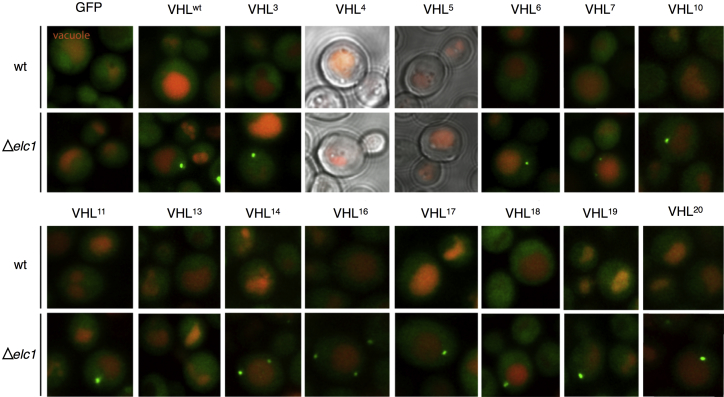
Cellular Phenotype Resulting from Elc1 Knockout Fluorescently tagged VHL mutants display a distinct cellular phenotype when expressed in the Δelc1 strain compared with the wild-type strain. Different panels show different mutant versions of VHL in both the wild-type (top) and Δelc1 (bottom) strains, with green representing the fluorescent protein fusion construct and red the vacuole. VHL^4^ and VHL^5^ could not be detected in the cell at appreciable quantities, and only GFP alone and VHL^13^ escaped the puncta phenotype characterized by the other VHL forms.

Having quantified fluorescent levels, we then looked more closely at the cell biological phenotypes of the different mutants. Further observation of the behavior of different VHL mutants in the Δ*elc1* environment revealed clear puncta formation for most mutants, including VHL^19^, in the absence of the yeast homolog of elongin C (Figure 6). Puncta formation is linked to different aspects of the PQC system, including differential processing of terminally misfolded proteins and as a response to external and internal stress (Amen and Kaganovich, 2015; Narayanaswamy et al., 2009). Elongin C is itself part of the polyubiquitination process, functioning as a component of an E3 ubiquitin ligase complex in both yeast and mammals and therefore directly contributing to PQC systems through its participation in ubiquitin-mediated degradation pathways (Lisztwan et al., 1999; Ramsey et al., 2004). Intriguingly VHL^13^ (which had also exhibited high fluorescence in the original flow cytometry experiment) escaped this cellular phenotype, remaining diffuse throughout the cell. This VHL sequence is the only one tested that changed a residue in a known chaperonin binding site, indicating that this mutation might be able to escape PQC recognition and avoid being sequestered in puncta before degradation. This result is certainly consistent with our original hypothesis that the higher fluorescence of VHL^13^ was due to altered chaperonin interaction.

## Discussion

The ability of a cell to recognize aberrant proteins and target them for either refolding or destruction is crucial to maintaining protein homeostasis and preventing pathological aggregation. At the same time, allosteric interactions caused by relatively minor mutational changes can have a large impact on how a protein is categorized by the PQC system. Consequently, an improved understanding of how potential PQC substrates may be driven between qualitatively different conformational states by various perturbations is essential to a full understanding of how the cell maintains proteostasis.

A persistent challenge in studying many PQC substrates is that by nature they tend to be structurally disordered and are therefore not easily amenable to traditional methods of in vitro structural characterization (Oldfield and Dunker, 2014). As a result, there is an opportunity for computational methods to play a central role in providing valuable structural information that can be used in combination with experimental results to better understand how the PQC fate of misfolding-prone proteins may be affected by mutation or stress. This study presents a novel method of integrating computational biophysics techniques to gain an experimentally testable glimpse into how marginal stability affects protein interactions with the PQC machinery. This approach of using in silico results to guide in vivo experiments, and vice versa, demonstrates that both approaches can be used in tandem to yield better explanations for these biophysical phenomena.

In particular, due to its marginal stability, VHL has been used as a model substrate for chaperone proteins involved in assisted folding pathways as well as for studying PQC systems. Hsp90, Hsp70, and TRiC have all been shown to interact with VHL in yeast, with the chaperonin TRiC potentially functioning as a holdase for containing nascent VHL until it can bind to its elongin cofactors (Feldman et al., 1999). Although TRiC has not been directly implicated in the degradation processes, some mutant forms of VHL have also been shown to be able to refold after denaturing events in a chaperonin-independent way in vitro but not in vivo (Feldman et al., 2003; Yang et al., 2013).

In this study, we present 20 novel pair-swap mutations of VHL that switch the position of two residues in the sequence and examine the protein products in yeast for a measure of their ability to escape rapid degradation by the cell. In particular, two mutations caused VHL to be detected in much larger amounts than that of transformed wild-type VHL: swapping the valine and glycine residues at positions 155 and 19, respectively (VHL^13^), and swapping the leucine and glutamic acid residues at positions 201 and 173 (VHL^19^). The first mutant directly affected a chaperone binding site, while the second did not mutate residues in any known chaperone or cofactor interaction motifs.

Initially, these results were surprising because both stabilizing mutations were chosen randomly as part of the control group, while the test group of ten mutants designed to exhibit more stability actually produced less stable behavior on average. The underlying rationale behind choosing this latter group of mutants focused on predicting how easily a protein structure could access different structures at a low energy, with the idea that sequences displaying the least variability in predicted structure also might indicate increased thermodynamic stability and decreased degradation by the PQC system. The behavior of these mutants in vivo illustrates the difficulty in teasing apart the mechanisms of PQC response for marginally stable proteins. In the case of the ten designed mutants, the lower than expected observed amounts present in the cell may have been indicative that the mutated sequences were kinetically trapped in one configuration that was more easily identified by the PQC system. An estimate between the random and non-random sets bounded the probability of observing no highly fluorescent hits in the non-random set while obtaining two or more hits in the random control set at a maximum value slightly below 0.1. It is possible that the structural variability calculations actually predicted the opposite effect than originally thought: namely, lower ability to persist in the cell rather than higher thermodynamic stability. In this case, our designed parameter to maintain protein structure despite small energetic fluctuations may have made these mutants more susceptible to degradation, underscoring the importance of examining how protein conformational changes can affect the outcome of PQC interactions. Future sequence design protocols along the lines of our initial attempt should therefore be modified to include not only the effect of structural variability but also the importance of maintaining resemblance to a native fold that exposes the right parts of the chain on the surface.

To explain the two unexpectedly stable hits in the random control sample, the biophysical burial mode model was used to examine each of the 20 mutated sequences. These structural predictions were also compared with the wild-type sequence and its experimentally determined structure. Strikingly, the burial mode model predicted that the mutated sequence of VHL^19^ would fold such that it would bury known hydrophobic chaperone interaction sites while exposing a motif known to be necessary and sufficient for binding to a homolog of its human cofactor elongin C in yeast (Figure 3). The two chaperone interaction sites have been identified as binding to TRiC, with the first motif also similar to the known Hsp70 recognition motif of a short hydrophobic sequence flanked by charged residues (Rüdiger et al., 1997). Thus, one possible effect of the VHL^19^ mutation could be to suppress interaction between the VHL protein and the PQC machinery. Even more intriguingly, the burial mode modeling also points to the possibility that this mutant form of VHL is stabilized because of interactions with a cofactor homolog that does not stabilize the wild-type sequence. The cofactor elongin C exists in yeast despite the absence of other VHL cofactors, but previous studies had indicated that it does not increase the half-life of wild-type VHL (McClellan et al., 2005). However, when we introduced VHL^19^ into a yeast strain lacking the elongin C homolog, levels of mutant VHL were decreased to that of the wild-type protein, providing compelling experimental evidence in support of the hypothesis that the yeast homolog of elongin C does substitute for the effect of the human cofactor as predicted and stabilizes the VHL^19^ mutant. This hypothesized interaction between the elongin cofactor homolog Elc1 and VHL in yeast was further supported when the different mutants were examined by microscopy (Figure 6), since the absence of Elc1 gives rise to a new quality control phenotype in wild-type and most mutated forms of VHL.

Using VHL as a model protein, the burial model has been shown to be capable of providing explanations of the relative likelihood of a mutated protein being degraded based on its ability to adopt different conformational states. Furthermore, this predictive capability was able to scan a large set of sequences in a relatively short time, providing rapid structural information about all possible mutations to narrow and refine experimental studies. The effectiveness of the burial mode model in explaining structural changes in mutant forms of VHL has significant implications for the underlying physical driving forces behind VHL stability. The model predicts structural change by calculating the trade-off between hydrophobic and steric effects; thus, our findings point to the possibility that the marginal stability of VHL originates in a fluctuating balance struck among three distinct stretches of moderately hydrophobic sequence (Box 1, Box2, and the ELC-binding region) that compete for limited space in the packed hydrophobic core of the protein.

Meanwhile, the elevated levels of VHL^13^, V155-G19, that we observed are more likely to have been the result of altered interaction with chaperones that directly followed from changes to known chaperone binding sites. The fact that this mutant was alone in escaping the puncta phenotype observed by microscopy in other VHL variants in the Δ*elc1* strain further illustrates the complex interactions that characterize cellular PQC systems involving both segregation and degradation of terminally misfolded proteins.

Stabilization both through modulation of chaperone interactions and through cooperative interactions with binding partners is implicated for VHL in determining how the PQC responds to conformational variants. Past computational work has underlined the importance of allosteric conformational change to VHL structure and function (Liu and Nussinov, 2008). Our findings confirm the importance of these effects specifically in connection to cofactor binding. Furthermore, this analysis also raises the question of whether or not the susceptibility of a marginally stable protein to adopting different structural forms might be advantageous to the cell, since this differential folding pattern based on small perturbations is directly coupled to PQC response. For example, an enhanced sensitivity to mutation can allow an additional level of cellular modulation of the functional fold of a protein, or can contribute to the enhanced ability of a protein to rapidly traverse an evolutionary landscape to find potential new folds.

Expanding our computational and experimental analysis to other proteins besides VHL may also prove informative in how the PQC responds to other marginally stable proteins, and in elucidating the importance of the structural flexibility of such a protein in a cellular context. The well-characterized p53 protein is similar to VHL in that it is small enough for burial trace analysis (393 amino acids), includes an N-terminal intrinsically disordered region, shows low thermodynamic and kinetic stability, and is characterized by a large number (>1,000) of cancer-related mutations found in humans (Joerger and Fersht, 2007). In addition, a recent study has indicated that p53 interacts with the same chaperonin, TRiC, that is involved in VHL folding (Trinidad et al., 2013). The similarity of this protein to VHL, in terms of both its functional properties and its clinical importance, makes it an enticing target for burial analysis. Further exploration of the structural basis of misfolded protein recognition, including of client tumor suppressors like VHL and p53, can give new insights into how cells maintain proteostasis and what structural mechanisms can drive qualitative shifts in the PQC fates of marginally stable proteins on the brink of structural disorder.

## Experimental Procedures

### Yeast Strains, Growth Conditions, and Materials

Yeast growth, media preparation, and manipulations were performed according to standard protocols (Adams et al., 1997). The strains used are listed in Table 2.

Plasmids used in this study are summarized in Table 3. VHL gene was fused to GFP or DDR2 and was expressed under the control of a galactose-regulated promoter (Gal1p); nls-TFP was used as a nuclear marker and expressed under GAL10p.

### Microscopy

For imaging, yeast cells were grown on galactose-containing medium to middle log phase and seeded on concanavalin A (Sigma)-coated four-well microscope plates (IBIDI). Confocal 3D images were acquired using a dual point-scanning Nikon A1R-si microscope equipped with a PInano Piezo stage (MCL), using a 60× PlanApo VC oil objective NA 1.40. Calculations of the fluorescence intensity and image processing were performed using NIS-Elements software.

### Single Cell Degradation Assay

Cells were grown as described earlier. The expression of GFP-VHL variants was induced by switching to galactose-containing medium for 6 hr. The degradation was calculated as a decrease in the green fluorescence intensity after cells were transferred to glucose medium (time 0). The fluorescence intensity of single cells was calculated using NIS software. All data points were normalized to GFP fluorescence decay under the same conditions.

### Fluorescence-Activated Cell Sorting Analysis

Yeast strains were grown on selection medium for 2 days, diluted twice a day to log phase, and on the third day, the fluorescence intensity of the DDR2 tag fused to VHL was analyzed by fluorescence-activated cell sorting with a 488 laser using BD FACSDiva (BD Biosciences) software.

### Mutagenesis

Different VHL mutants were cloned using a restriction-free cloning protocol as described by Van den Ent and Löwe (2006). Briefly, primers were designed according to the desired mutation and used for the first PCR reaction to amplify a mega primer carrying two mutations on the VHL sequence. Mega primers were used for a second PCR reaction to amplify the whole pDK5 plasmid with the desired two mutations using high-fidelity DNA polymerase (KAPA KR0370). Dpn1 digestion was used after the second PCR to get rid of the old methylated plasmids leaving only the new mutated plasmids. VHL mutants were then subcloned to pRS plasmids for fluorescence-activated cell sorting analysis.

### Statistical Analysis

To compare the designed mutants with the control group, we estimated our probability of the result arising from chance by calculating the number of hits (fluorescence levels above a threshold T) in both the design and control group:

Max(P(nohits>Tindesigned|f)×P(2ormorehits>Tincontrol|f))

where each probability is calculated as a binomial distribution arising from f, the underlying fraction of hits expected to be seen above the threshold. For the calculation in this study, the threshold was set at 60%.

## Author Contributions

K.B. and J.L.E. conducted all computational modeling and wrote the manuscript. A.A. created Dendra-tagged mutants and performed the flow cytometry experiments and T.A. created GFP-tagged constructs and performed fluorescence microscopy, including knockout studies. J.L.E. and D.K. oversaw all studies and helped formulate the experimental design. All authors helped edit the manuscript.

## Figures and Tables

**Table 1:** List of Mutants that Were Created Experimentally: Half Were Chosen Based on a Model Parameter and Half Were Chosen Randomly

Low Structural Variability Mutations	Random Mutations
1. K171 P61	11. E173 K196
2. R176 G14	12. E70 G19
3. V20 R3	13. V155 G19
4. P99 V74	14. T124 K159
5. V66 P40	15. T157 V194
6. K171 P5	16. D28 A56
7. V20 R4	17. G93 V181
8. R167 A11	18. R69 S168
9. C162 N7	19. L201 E173
10. Q203 N109	20. S183 C162

**Table 2:** Strains Used in this Study

Strain	Genotype	Reference
BY4741	BY4741 *MATa his3del0 leu2del0 met15del0 ura3del0*	Brachmann et al., 1998
BY4741 Δ*elc1*	BY4741 *MATa his3del0 leu2del0 met15del0 ura3del0 elc1::KANMX*	Winzeler et al., 1999
BY4741 *GFP*	BY4741 *his3::GALp-GFP -HIS3*	This study
BY4741 *GFP-VHL* (wild-type or mutants)	BY4741 *his3::GALp-GFP-VHL*^*(wt or mutants)*^*-HIS3*	This study

**Table 3:** Plasmids Used in this Study

Plasmid	Description	Reference
pDK399	pESC-*LEU-ELONGIN B/C*	This study
pDK2-22	pRS303-*GAL1p GFP GAL10p nls-TFP*	This study
pDK2-(2-21)	pRS303 *GAL1p GFP-VHL*^*wt or mutants*^*GAL10p nls-TFP*	This study
pDK5 (1-21)	pESC-*URA GAL1p GFP-VHL*^*wt or mutants*^*GAL10p nls-TFP*	This study
pDK437	pRS316 GPDp *DDR*2	This study
pDK437 (2-21)	pRS316 GPDp *DDR2 -VHL*^*wt or mutants*^	This study

## Supplemental Information

Document S1. Figures S1 and S2

Document S2. Article plus Supplemental Information
