# Supplementary material for: Structural Basis for Modulation of Quality Control Fate in a Marginally Stable Protein
Source: Structure. 2015 Jul 7;23(7):1169–78. doi: 10.1016/j.str.2015.04.015 (PMC4509718; doi:10.1016/j.str.2015.04.015)
Supplement: Document S1. Figures S1 and S2 [file mmc1.pdf]

Structure, Volume *23*

Supplemental Information

Structural Basis for Modulation of Quality Control Fate in a Marginally Stable Protein

Kelly P. Brock, Ayelet-chen Abraham, Triana Amen, Daniel Kaganovich, and Jeremy L. England

Supplemental Figures

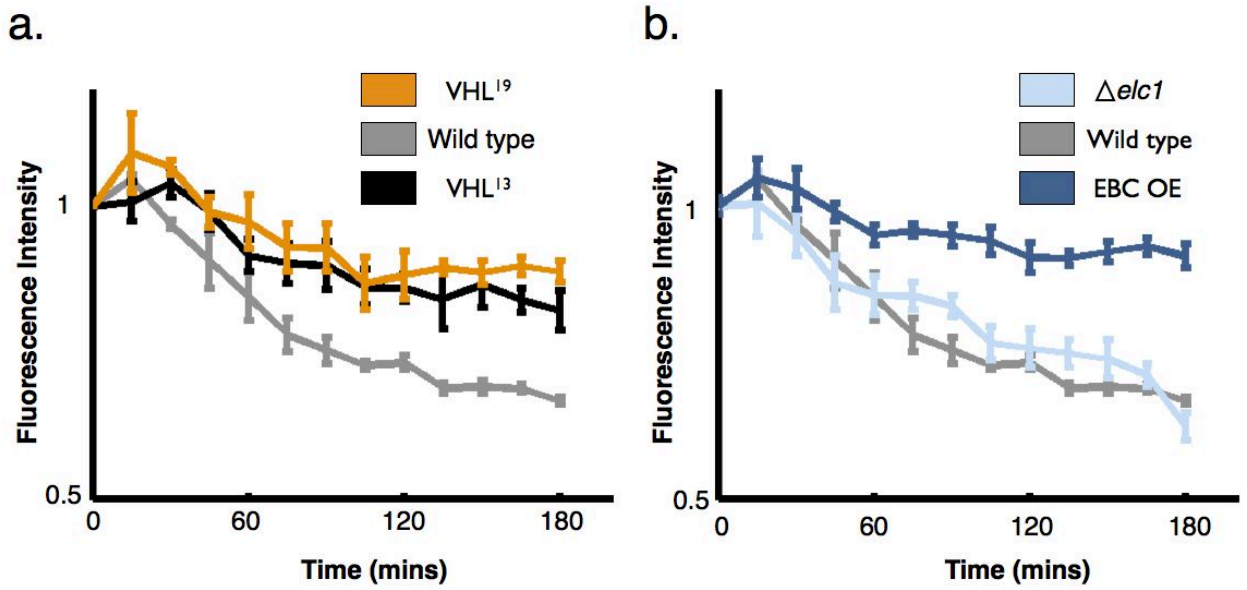

Figure S1, related to Figure 2. Degradation curves for VHL. *(a) Degradation curves are shown for the two most stable mutations, VHL^{19} (orange) and VHL^{13} (black), and wildtype sequence (grey). Both mutated sequences exhibit slower degradation compared to the wildtype. Curves are normalized to GFP degradation as well as initial values. (b) The degradation curves for the wildtype sequence under normal (grey), elc1 knockout (light blue), and human elongin BC overexpression (dark blue) are shown, using the same normalization scheme as in (a). Error bars for (a) and (b) are standard error over a triplicate experiment.*

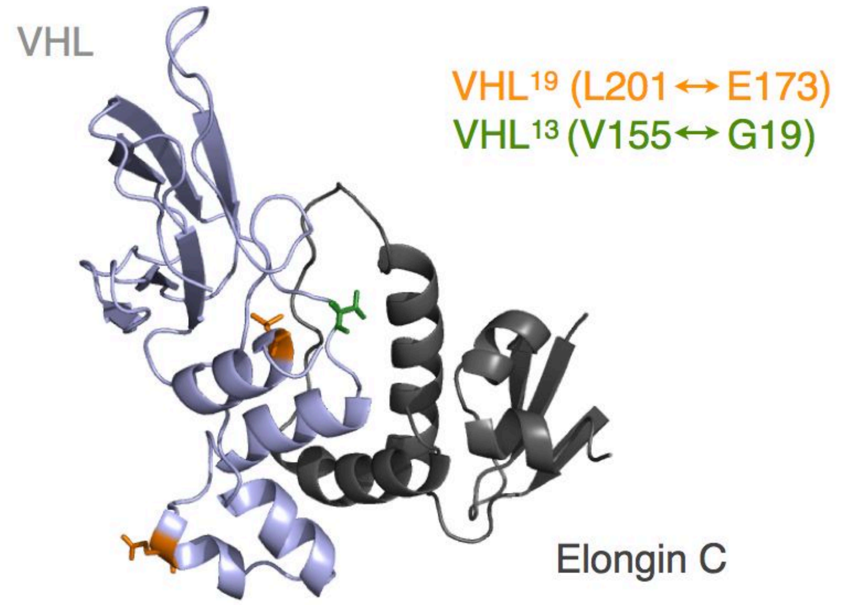

Figure S2, related to Figure 3. Positions of relevant mutations of VHL.

The crystal structure of VHL (light grey) with its binding partner elongin C (dark grey) is shown, with the mutated residues for VHL19 (orange) and VHL13 (green) . The second mutation in VHL13, at position 19, falls in a disordered region that has not been crystallized.
